# Supplementary material for: Structural basis of phosphorylation-independent nuclear import of CIRBP by TNPO3
Source: Nat Commun. 2025 May 14;16:4456. doi: 10.1038/s41467-025-59802-2 (PMC12075686; doi:10.1038/s41467-025-59802-2)
Supplement: Supplementary file 1 — Supplementary Information [file 41467_2025_59802_MOESM1_ESM.pdf]

Supplementary Information for
Structural basis of phosphorylation-independent nuclear import of CIRBP by TNPO3

Qishun Zhou[1,7], Theo Sagmeister[2], Saskia Hutten[3], Benjamin Bourgeois[1], Tea Pavkov-Keller[2,4,5], Dorothee Dormann[3,6], Tobias Madl*[1,5].
1. Research Unit Integrative Structural Biology, Medicinal Chemistry, Otto Loewi Research Center, Medical University of Graz, Graz, Austria.
2. Institute of Molecular Biosciences, University of Graz, Graz, Austria.
3. Johannes Gutenberg Universität Mainz, Institute of Molecular Physiology, Mainz, Germany.
4. Field of Excellence BioHealth, University of Graz, Graz, Austria.
5. BioTechMed-Graz, Graz, Austria.
6. Institute of Molecular Biology (IMB) Mainz, Mainz, Germany.
7. Present address: Institut Pasteur, Université Paris Cité, CNRS UMR3528, Bacterial Transmembrane Systems Unit, Paris, France.
*Tobias Madl is the corresponding author.

***Correspondence should be addressed to:**
Tobias Madl (lead contact)
Phone: (+43-316) 385-72120
Fax: (+43-316) 385-79622
E-Mail: tobias.madl@medunigraz.at

This PDF file includes:

Supplementary Figures 1 to 7
Supplementary Tables 1 to 2
Supplementary References

Other supporting materials for this manuscript include the following:

Supplementary Data 1 to 4

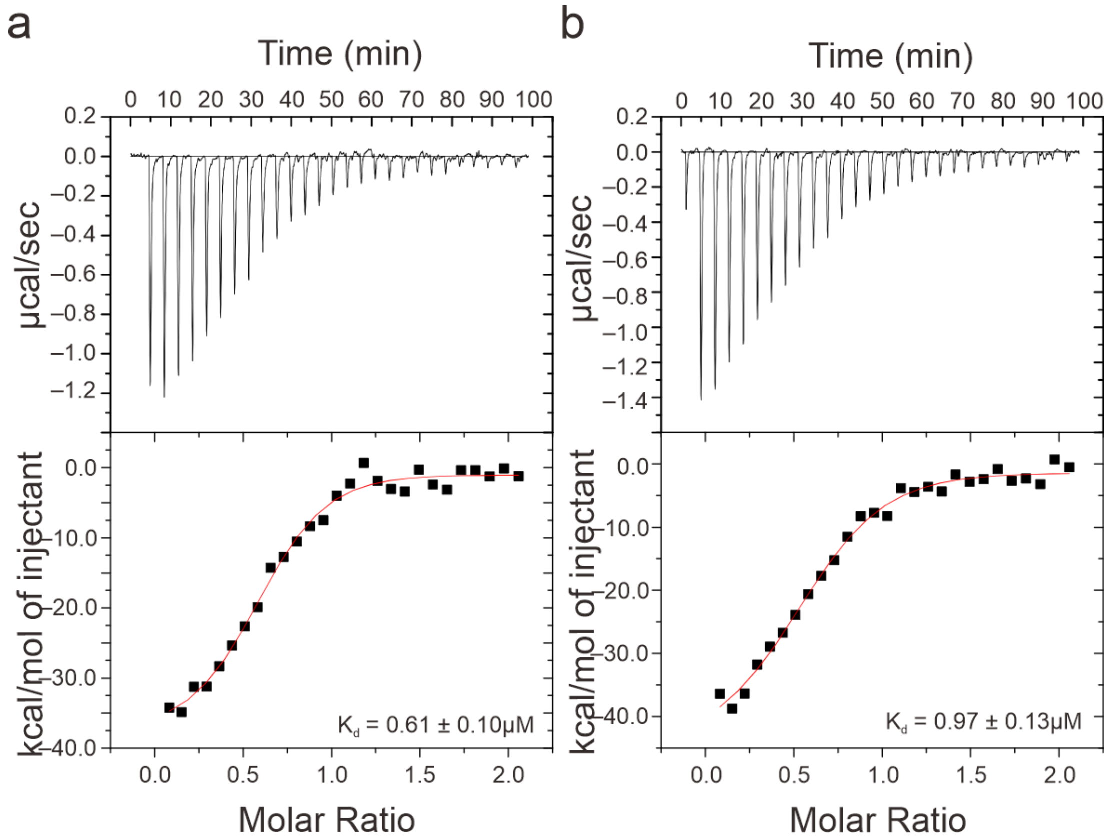

Supplementary Figure 1. ITC data for TNPO3 and TNPO3 C511A binding to recombinant CIRBP^{RSY}. (a) Titration of 100 µM CIRBP^{RSY} (137-172) into 10 µM of TNPO3 WT. (b) Titration of 100 µM CIRBP^{RSY} (137-172) into 10 µM of TNPO3 C511A. The reported errors correspond to the SD of the fit.

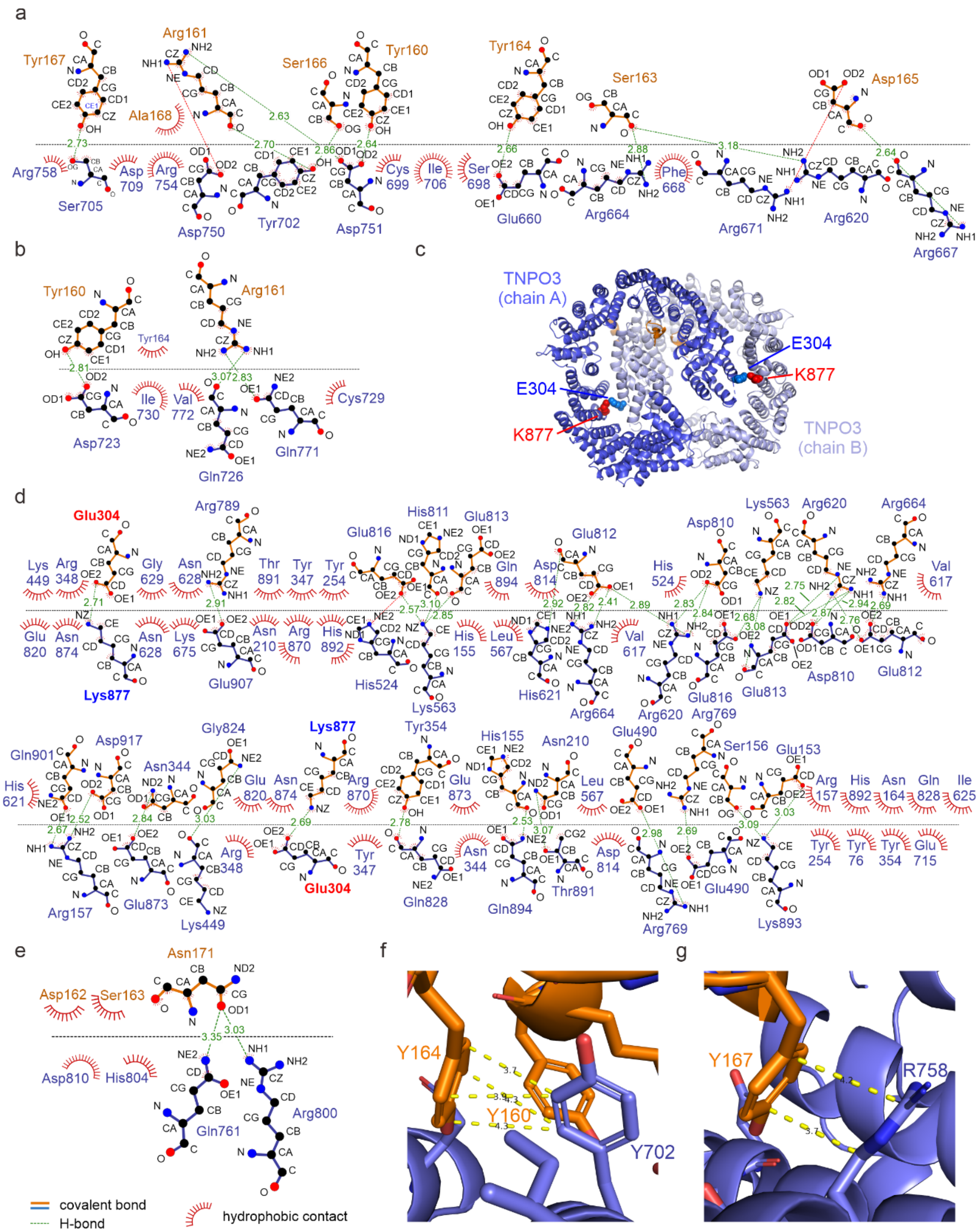

Supplementary Figure 2. Schematic representation of crystal contacts (PDB: 8CMK). (a) Plot corresponding to the TNPO3-CIRBP^{RSY} interactions identified from the second TNPO3 molecule in the dimer (below dotted line, blue) and its corresponding ligand CIRBP^{RSY} (above dotted line, orange). (b) Plot of intermolecular contacts between chain B (below dotted line, blue) of TNPO3 and chain E (above dotted line, orange) of CIRBP^{RSY} in the crystal structure. These interactions are putatively unspecific and are through the external surface of TNPO3. (c) Global view of the localization of the potential salt bridge formed between E304 and K877. (d) Plot of intermolecular

contacts between chain A (below dotted line, blue) and chain B (above dotted line, orange) of TNPO3 in the dimer. E304 and K877 are highlighted. (e) Plot of intermolecular contacts between chain B (below dotted line, blue) of TNPO3 and chain C (above dotted line, orange) of CIRBP^{RSY} in the crystal structure. The contact maps were generated with LigPlot+[1,2], and color and font sizes were adapted to match the color codes used in the manuscript. (f) Image showing aromatic side chains of TNPO3 Y702 (chain A, blue) and CIRBP Y164 (chain C, orange). The two aromatic cycles are perpendicular, indicating a π-stacking. (g) Image showing aromatic side chains of TNPO3 R758 (chain A, blue) and CIRBP Y167 (chain C, orange). The two aromatic cycles are in parallel, indicating a cation-π interaction.

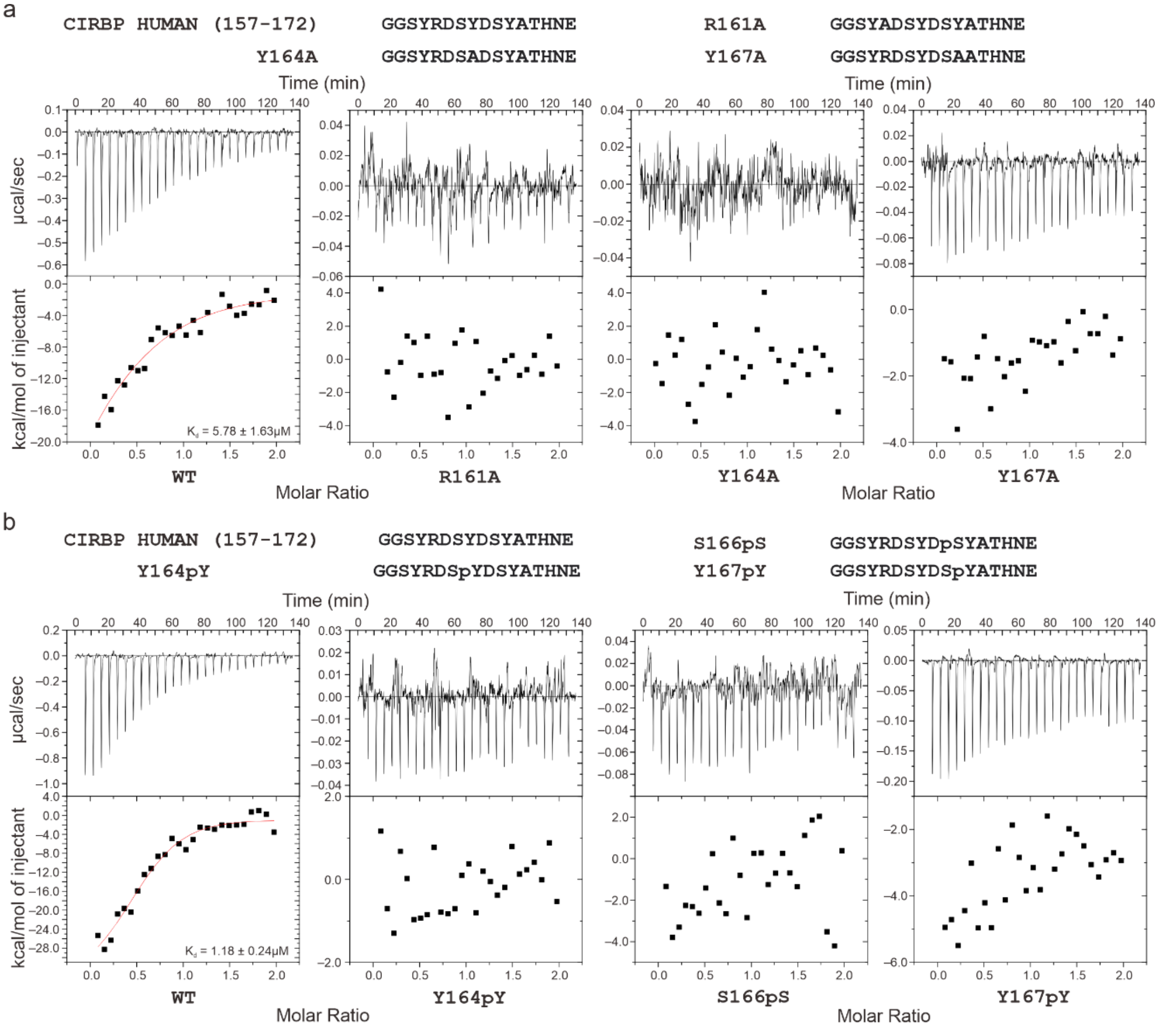

Supplementary Figure 3. ITC data for TNPO3 C511A with different variants of peptides derived from CIRBP^{RSY}. (a) Titration of 100 μM mCIRBP^{RSY} (157-172, left panel 1), R161A mutant of mCIRBP^{RSY} (157-172, left panel 2), Y164A mutant of mCIRBP^{RSY} (157-172, left panel 3) and Y167A mutant of mCIRBP^{RSY} (157-172, right panel) into 10 μM of TNPO3 C511A. (b) Titration of 100 μM mCIRBP^{RSY} (157-172, left panel 1) and the three phosphorylated mCIRBP^{RSY}, including Y164pY (left panel 2), S166pS (left panel 3) and Y167pY (right panel) into 10 μM of TNPO3 C511A. All reported errors correspond to the SD of the fit.

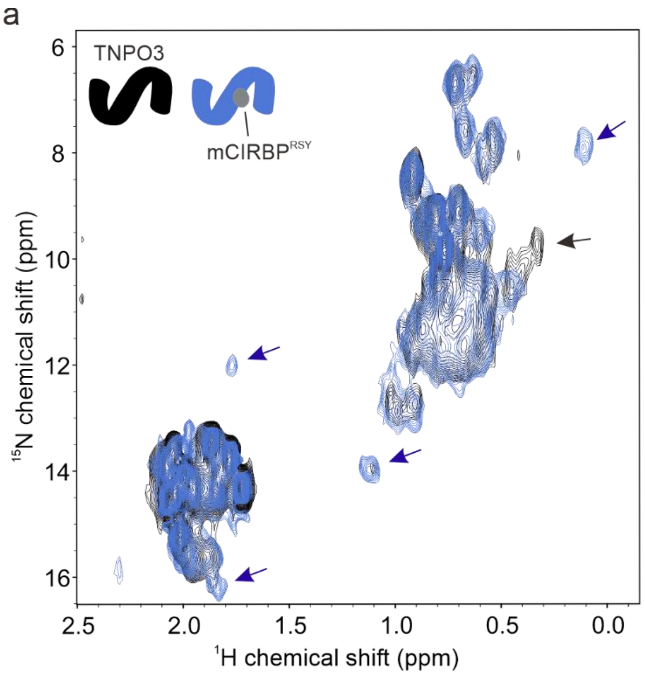

Supplementary Figure 4. Interaction between TNPO3 and mCIRBP. (a) ^{1}H - ^{13}C HMQC NMR spectra of 100 µM of [IM-methyl ^{13}C] labeled TNPO3 alone (black) and upon adding 1eq of mCIRBP^{RSY} (C-terminal CIRBP peptide, residues 157-172, blue). Peaks that appeared upon addition of ligand are indicated by blue arrows, whereas disappeared ones are indicated by black arrows.

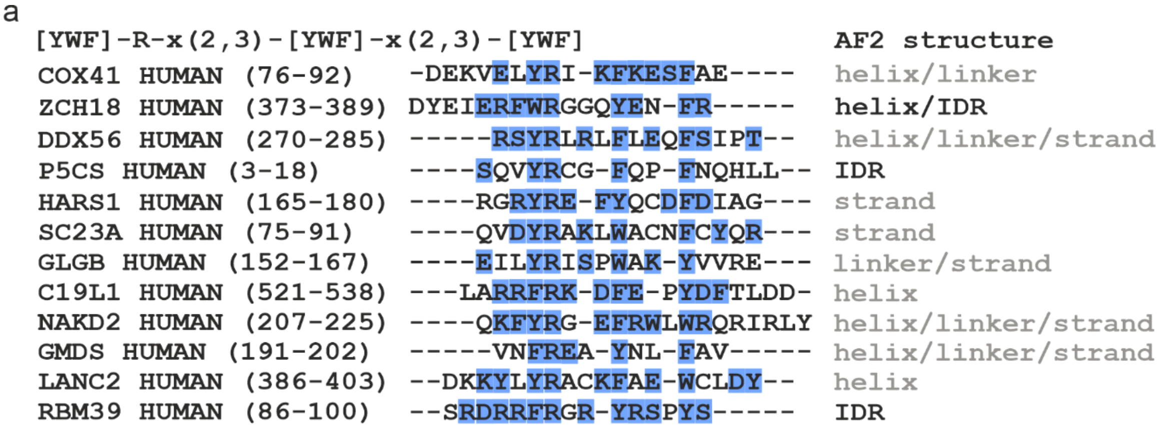

Supplementary Figure 5. List of the 12 potential TNPO3 cargos that contain [YWF]-R-x(2,3)-[YWF]-x(2,3)-[YWF] motif. These proteins have been identified as TNPO3 cargos in the previous study[3], their secondary structure refer to the AlphaFold2 structures available from UniProt[4-6]. Among the 12 proteins, nuclear proteins are ZCH18, DDX56, LANC2, and RBM39.

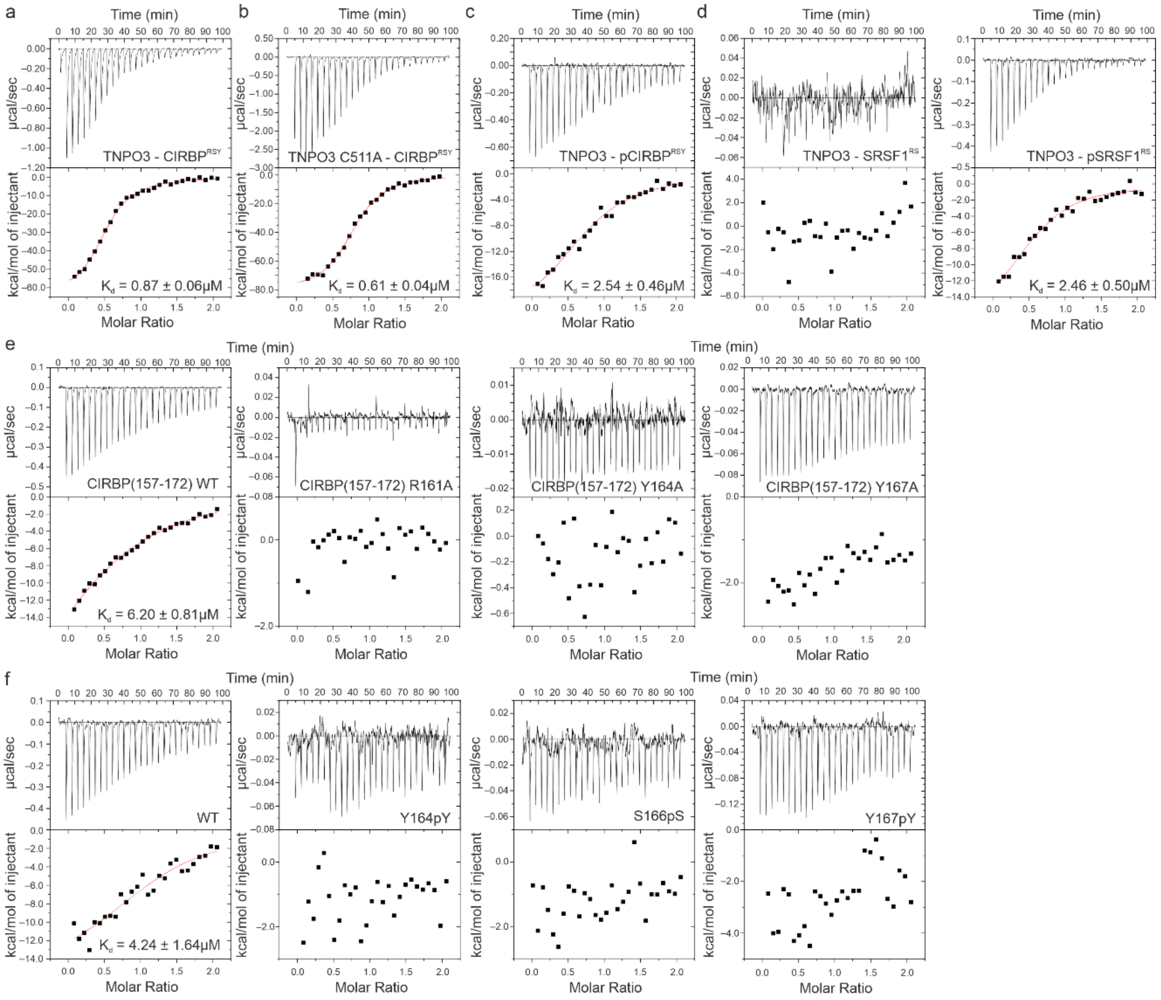

Supplementary Figure 6. Replicate ITC data for TNPO3 WT/C511A binding to target peptides. (a-b) ITC curve showing titrations of 100 µM CIRBP^{RSY} into 10 µM of (a) TNPO3 WT and (b) TNPO3 C511A mutant. (c) ITC curve showing titration of 100 µM phosphorylated pCIRBP^{RSY} into 10 µM of TNPO3 WT. (d) ITC curve showing titrations of 100 µM SRSF1^{RS} (left panel) and phosphorylated pSRSF1^{RS} (right panel) into 10 µM TNPO3 WT. (e) ITC curve of titrations of 100 µM peptides derived from CIRBP^{RSY} with alanine mutations into 10 µM of TNPO3 C511A. This includes mCIRBP^{RSY} (157-172, left panel 1), R161A mutant of mCIRBP^{RSY} (157-172, left panel 2), Y164A mutant of mCIRBP^{RSY} (157-172, left panel 3) and Y167A mutant of mCIRBP^{RSY} (157-172, right panel). (f) ITC curve of titrations of 100 µM peptides derived from CIRBP^{RSY} with phosphorylations into 10 µM of TNPO3 C511A. This includes mCIRBP^{RSY} (157-172, left panel 1), Y164pY variant of mCIRBP^{RSY} (157-172, left panel 2), S166pS variant of mCIRBP^{RSY} (157-172, left panel 3) and Y167pY variant of mCIRBP^{RSY} (157-172, right panel). The reported errors correspond to the SD of the fit.

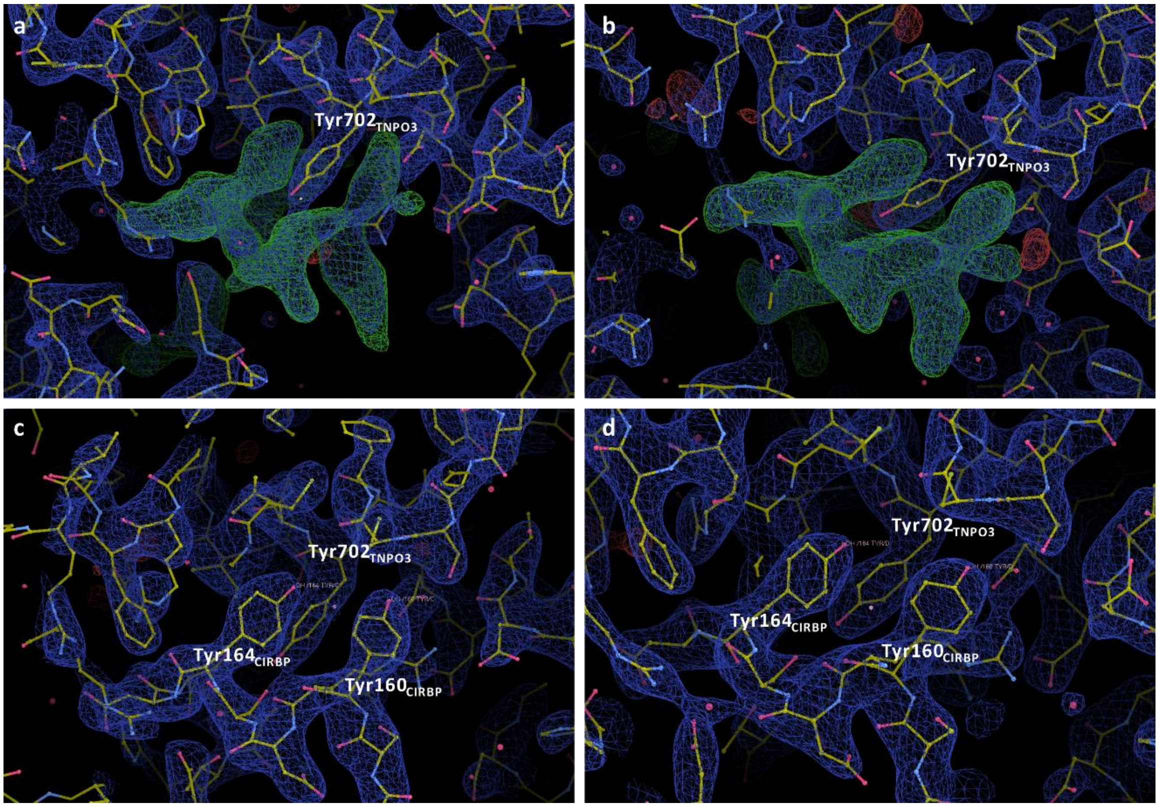

Supplementary Figure 7. Electron density of the crystal structure of TNPO3 bound to the ligand CIRBP (PDB: 8CMK). Panels a and b show the Fo–Fc (blue) and 2Fo–Fc (green for positive / red for negative) electron density maps for TNPO3 chains A and B, respectively, calculated without the CIRBP ligand (omit maps). In the region around $Tyr702_{TNPO3}$, the green positive Fo–Fc density clearly indicates the presence of the CIRBP ligand. Panels c and d show the 2Fo–Fc maps with CIRBP modeled for chains A and B of TNPO3, respectively. The electron density around key residues, $Tyr160_{CIRBP}$, $Tyr164_{CIRBP}$, and $Tyr702_{TNPO3}$, is well resolved. The contour levels for all maps are the same: 1.6σ for the 2Fo–Fc maps and 3.4σ for the Fo–Fc maps.

Supplementary Table 1. List of proteins and peptides, along with their affinities K_d, enthalpy changes (ΔH), and entropy changes (ΔS) binding to TNPO3.

Protein	Sequence	Mutation or PTM	TNPO3 binding K_d	ΔH (cal/mol)	ΔS (cal/mol/deg)
$CIRBP^{RSY}$ (137-172)	GSRDYYSSRSQSG-GYSDRSSGGSYRDSYDSYATHNE	N.A.	0.61 ± 0.10µM (rep1)<br>0.87 ± 0.06µM (rep2)	– 38480 ± 1354 (rep1)<br>– 65390 ± 1424 (rep2)	–101 (rep1)<br>–192 (rep2)
$CIRBP^{RSY}$ (137-172)	GSRDYYSSRSQSG-GYSDRSSGGSYRDSYDSYATHNE	N.A.	0.97 ± 0.13µM (rep1, binding to TNPO3 C511A)<br>0.61 ± 0.04µM (rep2)	– 45160 ± 1715 (rep1)<br>– 80500 ± 1237 (rep2)	–124 (rep1)<br>–242 (rep2)
$pCIRBP^{RSY}$(137-172)	Same as above, but with phosphoserine	phosphorylation	6.22 ± 1.19 µM (rep1)<br>2.54 ± 0.46 µM (rep2)	– 40560 ± 4350 (rep1)<br>– 22760 ± 1695 (rep2)	–112 (rep1)<br>–50.6 (rep2)
$SRSF1^{RS}$ (195-211)	DGPRSPSYGRSRSRSRS	N.A.	Not available (two replicates)	N.A.	N.A.
$pSRSF1^{RS}$ (195-211)	Same as above, but with phosphoserine	phosphorylation	2.49 ± 0.40 µM (rep1)<br>2.46 ± 0.50 µM (rep2)	– 10380 ± 500 (rep1)<br>– 17800 ± 1833 (rep2)	–9.12 (rep1)<br>–34.0 (rep2)
CIRBP (157-172) WT	GGSYRDSYDSYATHNE	N.A. (as a reference for alanine mutation)	5.78 ± 1.63 µM (rep1, binding to TNPO3 C511A)<br>6.20 ± 0.81 µM (rep2)	– 40350 ± 18230 (rep1)<br>– 24210 ± 2531 (rep2)	–111 (rep1)<br>–57.3 (rep2)
CIRBP (157-172) R161A	GGSYADSYDSYATHNE	To alanine	No binding (two replicates)	N.A.	N.A.
CIRBP (157-172) Y164A	GGSYRDSADSYATHNE	To alanine	No binding (two replicates)	N.A.	N.A.
CIRBP (157-172) Y167A	GGSYRDSYDSAATHNE	To alanine	Low affinity (two replicates)	N.A.	N.A.
CIRBP (157-172) WT	GGSYRDSYDSYATHNE	N.A. (as a reference for phosphorylation)	1.18 ± 0.24 µM (rep1, binding to TNPO3 C511A)<br>4.24 ± 1.64 µM (rep2)	– 34170 ± 2844 (rep1)<br>– 15680 ± 2206 (rep2)	–87.5 (rep1)<br>–27.7 (rep2)
CIRBP (157-172) Y164pY	GGSYRDSpYDSYATHNE	Tyrosine phosphorylation	No binding (two replicates)	N.A.	N.A.
CIRBP (157-172) S166pS	GGSYRDSYDpSYATHNE	Serine phosphorylation	No binding (two replicates)	N.A.	N.A.
CIRBP (157-172) Y167pY	GGSYRDSYDSpYATHNE	Tyrosine phosphorylation	Low affinity (two replicates)	N.A.	N.A.

Supplementary Table 2. List of AlphaFold2 multimer predictions of the binding complexes between TNPO3 and its putative NLSs.

Protein	UniProt entry	Residue number	Sequence	Predicted binding site	pTM*	ipTM
CIRBP	Q14011	157-172	GGSYRDSYDSYATHNE	Helix 14-17 (5/5 models)	0.877	0.658
SRSF1	Q07955	195-211	DGPRSPSYGRSRSRSRS	Helix 14-17 (5/5 models)	0.866	0.594
CPSF6	Q16630	520-528	YYRERSRER	Helix 14-17 (5/5 models)	0.874	0.722
RBMX/RMXL1	P38159/ Q96E39	276-291	PSGGSYRDSYESYGNS	Helix 14-17 (5/5 models)	0.881	0.763
AKAP8	O43823	143-157	HNPYRPSYSYDYEFD	Helix 14-17 (5/5 models)	0.871	0.579
CALU	O43852	125-141	SWDEYRNVTYGTYLDDP	Helix 15-16 (1/5 models)	0.869	0.504
CBPC2	Q5U5Z8	16-36	PYEDFMYRHLQYYGYFKAQRG	Diverged binding sites	0.869	0.402
CENPC	Q03188	391-404	TVNNYRSTKYEMYS	Helix 15-18 (5/5 models)	0.882	0.604
CLK4	Q9HAZ1	64-79	ERDYRDRRYVDEYRND	Helix 14-17 (5/5 models)	0.873	0.656
CSPP1	Q1MSJ5	543-557	ATNYRTPYDDAYYFY	Helix 14-17 (5/5 models)	0.882	0.575
DLGP3	O95886	734-749	QGQWAYREGYPLPYEP	Helix 15-18 (4/5 models)	0.876	0.46
FBLN5	Q9UBX5	73-87	NPVYRGPYSNPYSTP	Helix 15-18 (5/5 models)	0.875	0.486
FRM4A	Q9P2Q2	830-843	SQYRIKEYPLYIEG	Helix 15-17 (5/5 models)	0.881	0.564
F186A	A6NE01	2018-2035	HFTKYRTPVYQTPYTDER	Helix 14-18 (5/5 models)	0.873	0.52
GRIA3	P42263	877-891	YATYREGYNVYGTES	Helix 15-18 (5/5 models)	0.876	0.763
HNRL2	Q1KMD3	686-703	GYRNFYDRYRGDYDRFYG	Helix 15-18 (5/5 models)	0.875	0.564
HnRNPQ	O60506	471-487	GYDYHNYRGGYEDPYYG	Helix 14-18 (5/5 models)	0.882	0.603
HNRPR	O43390	476-492	GYDYHDYRGGYEDPYYG	Helix 15-18 (5/5 models)	0.881	0.613
IF4B	P23588	230-244	SDRYRDGYRDGYRDG	Helix 15-18 (5/5 models)	0.882	0.658
KDIS	Q9ULH0	1380-1395	QAEYRDAYREYIAQMS	Helix 15-18 (2/5 models)	0.87	0.44
KHDR1	Q07666	433-443	GAYREHPYGRY	Helix 15-18 (5/5 models)	0.876	0.673
KHDR2	Q5VWX1	339-349	GGYREHPYGRY	Helix 15-18 (5/5 models)	0.876	0.671
KHDR3	O75525	333-346	TAKGVYRDQPYGRY	Helix 15-18 (5/5 models)	0.875	0.632
KR161	A8MUX0	420-431	SYRPACYRPCYS	Helix 15-18 (5/5 models)	0.867	0.602
KR193	Q7Z4W3	64-78	YGCYRPSYYGGYGFS	Helix 14-17 (5/5 models)	0.866	0.534
KR212	Q3LI59	67-83	CCGYRPLCYRRCYSSCY	Helix 14-17 (5/5 models)	0.864	0.544
LYOX	P28300	197-211	GRYRPGYGTGYFQYG	Helix 15-18 (3/5 models)	0.872	0.519
RBBP6	Q7Z6E9	831-845	ERKYREWYEKYYKGY	Helix 15-18 (4/5 models)	0.864	0.563
RBM10	P98175	33-47	DHDYRDMDYRSYPRE	Helix 14-17 (5/5 models)	0.878	0.679
SC16A	O15027	1159-1172	YYYYRPLYDAYQPQ	Helix 15-17 (5/5 models)	0.871	0.529
SIAL	P21815	36-49	VFKYRPRYYLYKHA	Helix 15-17 (2/5 models)	0.867	0.549
SOX15	O60248	157-174	SRGFGYRPPSYSTAYLP	Diverged binding sites	0.864	0.384
SPTB2	Q01082	2138-2156	SPRVSYRSQTYQNYKNFNS	Helix 15-18 (5/5 models)	0.867	0.511
TBX10	O75333	319-333	PATYRPVTYQSLYSG	Diverged binding sites	0.869	0.406
ZN644	Q9H582	736-753	SHYLYRHKYENYRMIKKS	Helix 15-18 (2/5 models)	0.875	0.541
P5CS	P54886	3-20	SQVYRCGFQPFNQHLLPW	Helix 1-3 (5/5 models)	0.867	0.481
ZCH18	Q86VM9	371-390	YYDYEIERFWRGGQYENFRV	Helix 15-18 (5/5 models)	0.872	0.564
RBM39	Q14498	86-101	SRDRRFRGRYRSPYSG	Helix 14-17 (5/5 models)	0.871	0.552

*pTM and ipTM are the values corresponding to the rank 001 of the predicted structure with relaxation.

Supplementary References

1 Laskowski, R. A. & Swindells, M. B. LigPlot+: multiple ligand-protein interaction diagrams for drug discovery. *J Chem Inf Model* **51**, 2778-2786, doi:10.1021/ci200227u (2011).

2 Wallace, A. C., Laskowski, R. A. & Thornton, J. M. LIGPLOT: a program to generate schematic diagrams of protein-ligand interactions. *Protein Engineering, Design and Selection* **8**, 127-134, doi:10.1093/protein/8.2.127 (1995).

3 Kimura, M. *et al.* Extensive cargo identification reveals distinct biological roles of the 12 importin pathways. *eLife* **6**, e21184, doi:10.7554/eLife.21184 (2017).

4 Jumper, J. *et al.* Highly accurate protein structure prediction with AlphaFold. *Nature* **596**, 583-589, doi:10.1038/s41586-021-03819-2 (2021).

5 Varadi, M. *et al.* AlphaFold Protein Structure Database: massively expanding the structural coverage of protein-sequence space with high-accuracy models. *Nucleic Acids Research* **50**, D439-D444, doi:10.1093/nar/gkab1061 (2022).

6 The UniProt, C. UniProt: the Universal Protein Knowledgebase in 2023. *Nucleic Acids Research* **51**, D523-D531, doi:10.1093/nar/gkac1052 (2023).
