# Supplementary material for: Structural basis of phosphorylation-independent nuclear import of CIRBP by TNPO3
Source: Nat Commun. 2025 May 14;16:4456. doi: 10.1038/s41467-025-59802-2 (PMC12075686; doi:10.1038/s41467-025-59802-2)
Supplement: Supplementary file 2 — Description of Additional Supplementary Files [file 41467_2025_59802_MOESM2_ESM.pdf]

Description of Additional Supplementary Files

File name: Supplementary Data 1
Description: Lists of human proteins that contain a motif of three consecutive SR/RS repeats.

File name: Supplementary Data 2
Description: List of human proteins containing at least one Y-R-x(2,3)-Y-x(2,3)-Y motif (Datasheet 1) and the subset of these in which the motif is located within IDRs (Datasheet 2).

File name: Supplementary Data 3
Description: List of human proteins containing at least one [YWF]-R-x(2,3)-[YWF]-x(2,3)-[YWF] motif (Datasheet 1) and the subset previously identified as TNPO3 cargos in published studies (Datasheet 2).

File name: Supplementary Data 4
Description: List of human nuclear proteins downloaded directly from the UniProt database and dated to July 2024 (https://www.uniprot.org/).
