# Supplementary material for: Structural basis of phosphorylation-independent nuclear import of CIRBP by TNPO3
Source: Nat Commun. 2025 May 14;16:4456. doi: 10.1038/s41467-025-59802-2 (PMC12075686; doi:10.1038/s41467-025-59802-2)
Supplement: Supplementary file 7 — Reporting Summary [file 41467_2025_59802_MOESM7_ESM.pdf]

nature portfolio

Corresponding author(s): Tobias Madl

Last updated by author(s): Apr 25, 2025

# Reporting Summary

Nature Portfolio wishes to improve the reproducibility of the work that we publish. This form provides structure for consistency and transparency in reporting. For further information on Nature Portfolio policies, see our Editorial Policies and the Editorial Policy Checklist.

## Statistics

For all statistical analyses, confirm that the following items are present in the figure legend, table legend, main text, or Methods section.

| n/a | Confirmed | |
|---|---|---|
| ☐ | ☒ | The exact sample size (*n*) for each experimental group/condition, given as a discrete number and unit of measurement |
| ☐ | ☒ | A statement on whether measurements were taken from distinct samples or whether the same sample was measured repeatedly |
| ☒ | ☐ | The statistical test(s) used AND whether they are one- or two-sided<br>*Only common tests should be described solely by name; describe more complex techniques in the Methods section.* |
| ☒ | ☐ | A description of all covariates tested |
| ☒ | ☐ | A description of any assumptions or corrections, such as tests of normality and adjustment for multiple comparisons |
| ☐ | ☒ | A full description of the statistical parameters including central tendency (e.g. means) or other basic estimates (e.g. regression coefficient) AND variation (e.g. standard deviation) or associated estimates of uncertainty (e.g. confidence intervals) |
| ☒ | ☐ | For null hypothesis testing, the test statistic (e.g. *F*, *t*, *r*) with confidence intervals, effect sizes, degrees of freedom and *P* value noted<br>*Give P values as exact values whenever suitable.* |
| ☒ | ☐ | For Bayesian analysis, information on the choice of priors and Markov chain Monte Carlo settings |
| ☒ | ☐ | For hierarchical and complex designs, identification of the appropriate level for tests and full reporting of outcomes |
| ☒ | ☐ | Estimates of effect sizes (e.g. Cohen's *d*, Pearson's *r*), indicating how they were calculated |

*Our web collection on statistics for biologists contains articles on many of the points above.*

## Software and code

Policy information about availability of computer code

| | |
|---|---|
| Data collection | All hardware and equipment details are provided in the Materials and Methods section. NMR spectra were acquired using Bruker TopSpin, versions 4.1.3 and 4.3.0. X-ray diffraction data for crystal structure determination were collected at the PETRA III synchrotron-radiation facility (DESY, Hamburg, Germany) on February 5, 2021, using Beamline P11 and a DECTRIS EIGER2 X 16M detector at 100 K, with a wavelength of 1.03322 Å. Motif scanning was conducted using the ScanProsite tool, available through the Expasy PROSITE webserver (SIB Swiss Institute of Bioinformatics). Structural predictions of the TNPO3–ligand binding complex were generated using AlphaFold2, implemented via ColabFold v1.5. |
| Data analysis | NMR spectra have been processed and analysed using Bruker Topspin 4.3.0, Spectral exports for figure preparation were generated using UCSF Sparky version 3.114. Analysis of NMR spectra for the phosphorylation of CIRBPRSY was performed using CCPNMR Analysis version 2.5.2. Graphical data representations were created using GraphPad Prism 9.<br>Raw diffraction data of TNPO3-ligand crystal have been analysed by XDS (20230630) for indexing and integrating and data reduction was done with AIMLESS (0.7.7). The structure was solved with Phaser (2.8.3), and has been rebuilt and improved with Coot (v.0.9.8.93), as well as refined with REFMAC5. Ramachandran and rotamer outliers were improved within ChimeraX (v.1.7), and ISOLDE Plugin (v.1.6). The final refinement statistics were generated with Phenix (1.20.1). The final structure was analysed by PyMOL v2.5 and LigPlot+ v2.2.<br>Images of nuclear import assay has been analysed with Fiji/Image J (1.54) and StackReg Plugin, and quantitative data were plotted using GraphPad Prism 9.<br>Alphafold2-predicted structures have been analysed with PyMOL v2.5. |

For manuscripts utilizing custom algorithms or software that are central to the research but not yet described in published literature, software must be made available to editors and reviewers. We strongly encourage code deposition in a community repository (e.g. GitHub). See the Nature Portfolio guidelines for submitting code & software for further information.

# Data

Policy information about availability of data

All manuscripts must include a data availability statement. This statement should provide the following information, where applicable:

- Accession codes, unique identifiers, or web links for publicly available datasets
- A description of any restrictions on data availability
- For clinical datasets or third party data, please ensure that the statement adheres to our policy

The structural data used in this study include four Protein Data Bank (PDB) entries: 8CMK [https://doi.org/10.2210/pdb8CMK/pdb] (TNPO3 in complex with RSY region of CIRBP), 4C0O [https://doi.org/10.2210/pdb4C0O/pdb] (Transportin 3 in complex with phosphorylated ASF/SF2), and 6GX9 [https://doi.org/10.2210/pdb6GX9/pdb] (TNPO3 - CPSF6 RSLD complex), 4C0P [https://doi.org/10.2210/pdb4C0P/pdb] (Unliganded Transportin 3). The structure of the TNPO3-CIRBPRSY complex was generated in this study and deposited in the RCSB Protein Data Bank under the accession code 8CMK. 4C0O, 4C0P and 6GX9 are previously published structures and are properly cited. NMR chemical shift assignments were transferred from previously published data BMRB 28025 [https://dx.doi.org/10.13018/BMR28025] (human_CIRBP_138-172), which is also properly cited. Raw NMR data can be reproduced based on the protein preparation details in the Methods section. The ITC data generated in this study are provided in the Supplementary Information. Source data underlying the plot of CIRBP nuclear import and phosphorylation kinetics are provided in the Source Data file. AlphaFold2 structures generated in this study have been deposited in the Zenodo repository (https://zenodo.org, https://doi.org/10.5281/zenodo.15268248). Researchers seeking access to original datasets not publicly available may contact the corresponding author.

# Research involving human participants, their data, or biological material

Policy information about studies with human participants or human data. See also policy information about sex, gender (identity/presentation), and sexual orientation and race, ethnicity and racism.

| | |
|---|---|
| Reporting on sex and gender | N/A |
| Reporting on race, ethnicity, or other socially relevant groupings | N/A |
| Population characteristics | N/A |
| Recruitment | N/A |
| Ethics oversight | N/A |

Note that full information on the approval of the study protocol must also be provided in the manuscript.

# Field-specific reporting

Please select the one below that is the best fit for your research. If you are not sure, read the appropriate sections before making your selection.

[x] Life sciences [ ] Behavioural & social sciences [ ] Ecological, evolutionary & environmental sciences

For a reference copy of the document with all sections, see nature.com/documents/nr-reporting-summary-flat.pdf

# Life sciences study design

All studies must disclose on these points even when the disclosure is negative.

| | |
|---|---|
| Sample size | The sample size was determined based on standard practices in the field, taking into account data reproducibility and the ability to obtain meaningful statistical measures (mean and standard deviation). For live-cell imaging of nuclear import rates, the decision was based on prior experience and published data on this specific reporter protein (Bourgeois et al., 2020, PNAS). The sample size (n) for each experiment is indicated in the corresponding figure legends. |
| Data exclusions | Cells displaying stress granule (SG)-like structures for the respective reporter protein were excluded from the Fiji-based analysis, as SGs have been reported to interfere with nuclear import. Such cases were rare. |
| Replication | For live-cell imaging experiments, at least 40 cells per reporter/experiment were analysed and a total of 3 replicates (for CIRBP reporters) or 2 replicates (for empty control), respectively, were performed according to standard scientific practice. All the experiments for which replication is standard praxis in the field were repeated independently. The number of replicates and precise number of data points are mentioned in the respective figure legends. |
| Randomization | Randomization was not relevant to this study as this work involves in vitro characterization and no human participants or biological matter. Image acquisition and analysis was performed in a non-blinded, non -randomized fashion, as an effect by any unconscious bias of the researcher would not affect the results. |
| Blinding | Blinding was not relevant to this study as this work involves in vitro characterization and no human participants or biological matter, as mentioned above. |

# Reporting for specific materials, systems and methods

We require information from authors about some types of materials, experimental systems and methods used in many studies. Here, indicate whether each material, system or method listed is relevant to your study. If you are not sure if a list item applies to your research, read the appropriate section before selecting a response.

## Materials & experimental systems

| n/a | Involved in the study |
|---|---|
| ☒ | ☐ Antibodies |
| ☐ | ☒ Eukaryotic cell lines |
| ☒ | ☐ Palaeontology and archaeology |
| ☒ | ☐ Animals and other organisms |
| ☒ | ☐ Clinical data |
| ☒ | ☐ Dual use research of concern |
| ☒ | ☐ Plants |

## Methods

| n/a | Involved in the study |
|---|---|
| ☒ | ☐ ChIP-seq |
| ☒ | ☐ Flow cytometry |
| ☒ | ☐ MRI-based neuroimaging |

## Eukaryotic cell lines

Policy information about cell lines and Sex and Gender in Research

| | |
|---|---|
| Cell line source(s) | HeLa cells were provided by Marc-David Ruepp, KCL London |
| Authentication | The cell line was recently authenticated using the Eurofins Cell Line Authentication Services. |
| Mycoplasma contamination | All cell lines were negative for mycoplasma in our regular tests. |
| Commonly misidentified lines (See ICLAC register) | No commonly misidentified cell lines were used in this study |

## Plants

| | |
|---|---|
| Seed stocks | N/A |
| Novel plant genotypes | N/A |
| Authentication | N/A |
